# Supplementary material for: Evaluation of the Susceptibility of Lotus Seeds (Nelumbo nucifera Gaertn.) to Aspergillus flavus Infection and Aflatoxin Contamination
Source: Toxins (Basel). 2024 Jan 7;16(1):29. doi: 10.3390/toxins16010029 (PMC10820585; doi:10.3390/toxins16010029)
Supplement: Supplementary file 1 [file toxins-16-00029-s001.zip › toxins-2800431-supplementary.pdf]

Evaluation of the Susceptibility of Lotus Seeds (*Nelumbo nucifera* Gaertn.) to *Aspergillus flavus* Infection and Aflatoxin Contamination

Abdelrahman Elamin, Sharmin Sultana and Shohei Sakuda

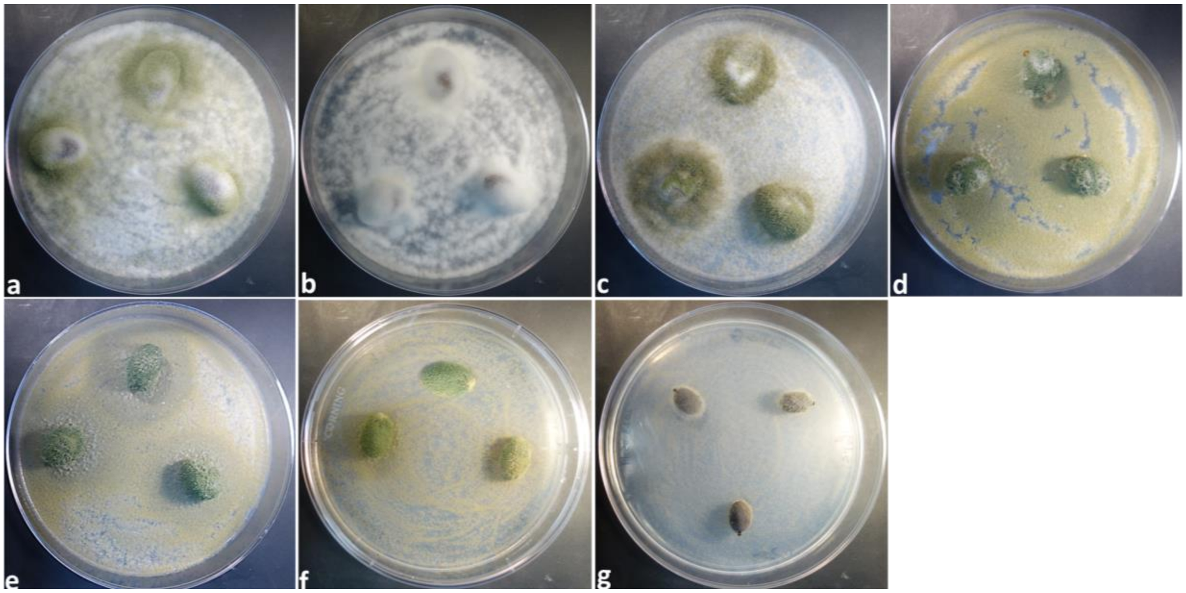

Figure S1. Artificial contamination of lotus seeds at different maturity (a–g) after 14 days incubation at 25°C. (a) lotus seeds of A stage. (b) lotus seeds of B stage. (c) lotus seeds of C stage. (d) lotus seeds of D stage. (e) lotus seeds of E stage. (f) lotus seeds of F stage. (g) lotus seeds of G stage.
